# Fat-Soluble Vitamins Deficiency in Pediatric Cholestasis: A Scoping Review

**DOI:** 10.3390/nu15112491

**Published:** 2023-05-26

**Authors:** Irene Degrassi, Ilaria Leonardi, Elisabetta Di Profio, Chiara Montanari, Gianvincenzo Zuccotti, Elvira Verduci

**Affiliations:** 1 Department of Paediatrics, Buzzi Children’s Hospital, 20154 Milan, Italy; elisabetta.diprofio@unimi.it (E.D.P.); chiara.montanari@unimi.it (C.M.); gianvincenzo.zuccotti@unimi.it (G.Z.);; 2 Department of Health Sciences, University of Milan, 20146 Milan, Italy; ilaria3leonardi@gmail.com; 3 Department of Biomedical and Clinical Sciences, University of Milan, 20157 Milan, Italy

**Keywords:** cholestasis, vitamins deficiency, malnutrition, retinol, cholecalciferol, vitamin K, tocopherol

## Abstract

Background: This review aims to identify the current indications and gaps in the management of fat-soluble vitamins in pediatric patients with cholestasis. Methods: A comprehensive review of the literature using PubMed, Scopus, Web of Science and Embase was performed. Two authors independently identified the most relevant studies published over the past 20 years up to February 2022, including original papers, narrative reviews, observational studies, clinical trials, systematic reviews and meta-analyses. The literature was screened, and preclinical studies about pathogenetic mechanisms were also included. Keywords searched for each fat-soluble vitamin (A, D, E and K), alone or in combination, were “cholestasis”, “chronic liver disease”, “biliary atresia”, “malnutrition” and “nutritional needs”. Studies published prior to the selected time range were searched manually and, when considered relevant, included within the list of references. Results: Eight hundred twenty-six articles were initially screened. From these, 48 studies were selected. A comparison of the recommended methods of supplementation for fat-soluble vitamins was then carried out. The causes of malabsorption were explained and current methods for defining deficiency and monitoring complications were summarized. Conclusions: According to the literature, children with cholestasis are at a higher risk of fat-soluble vitamin deficiency. Although there are general recommendations, the treatment for vitamin deficiency is not uniformly validated.

## 1. Introduction

In the pediatric population, cholestasis predominantly affects newborns and infants. It affects approximately 1 in 2500 full-term births, with a substantial percentage having a chronic course. It has been defined as “chronic cholestatic liver disease” (CCLD), which can lead to end-stage liver disease, hence requiring liver transplantation.

In the cholestatic diseases, impaired secretion of bile acids into the intestinal lumen and fat malabsorption induce a deficiency of fat-soluble vitamins (A, D, E and K). Absorption of these micronutrients requires the formation of micelles that solubilize liposoluble components in the intestine and facilitate their passive diffusion through the enterocyte. If the bile acid level in the intestinal lumen is below a critical micellar concentration, absorption of fat-soluble vitamins is impaired, thus leading to major complications, such as rickets, xerophthalmia and bleeding [1]. 

When cholestasis begins in childhood, it is crucial to ensure an adequate intake of vitamins, adjusting the dosages to the specific needs of the patient and avoiding the onset of side effects. In the literature, the indications for supplementation have not been standardized, although different routes of administration and wide dosage ranges have been published. The aim of this review is to summarize and compare the data about deficiency of fat-soluble vitamins in cholestatic pediatric patients (CPP) to understand the real incidence of the problem and to conduct a comparison on the current indications.

### 1.1. Incidence of Fat-Soluble Vitamin Deficiency in Cholestasis

Fat-soluble vitamin (FSV) deficiency is one of major nutritional problems of chronic cholestatic liver disease. Several studies report different vitamin A, E and D deficiencies in patients with chronic liver disease (CLD) and cholestasis [2,3] (Table 1). 

The incidence of vitamin deficiency was reported to be as high as 20–30%, in CPP [6], with even higher rates estimated in patients with biliary atresia [5]. According to a retrospective study [3], the prevalence of FSV deficiency in a pediatric population awaiting liver transplantation (166 children, including 126 with cholestasis) was 66.6%, 40.6% and 36.3%, respectively, of vitamin A, E and D, before liver transplantation [3]. A cross-sectional study in children with BA found that vitamin A and E deficiencies were more frequent in patients with cholestatic disorders (77.2% and 50.9%) than in those with non-cholestatic disorders (36.8% and 10.5%, *p* < 0.001), whereas vitamin D deficiency was similar between CPP and non-cholestatic patients (37.6% vs. 30.8%, *p* = 0.441) [2].

### 1.2. Indirect Laboratory Markers of Fat-Soluble Vitamins Deficiency

Vitamin storages present at birth are rapidly depleted; hence, if cholestasis starts in the first months of life, the first biochemical and clinical signs of fat-soluble vitamin deficiency may appear before the first year of life [7,8]. Therefore, from the earliest stage of neonatal cholestasis, infants should be screened regularly for monitoring their vitamin status, preventing deficiencies, adjusting dosages to patient needs and intervening promptly in case of any side effects [1]. It was hypothesized that the marker with the best correlation with vitamin deficiency was the serum bile acid (SBA) assay [1]. The efficacy of the serum bile acid assay versus total serum bilirubin (TB) levels was compared in predicting biochemical FVS deficiency in 92 infants with biliary atresia [1]. In contrast to the initial study hypothesis, total serum bilirubin (TB) was found to correlate better with fat-soluble vitamin deficiency than serum bile acid dosage. From a practical standpoint, the dosage of bilirubin is more easily performed and less expensive than the serum bile acid assay. In fact, fat-soluble deficiency was inversely related to total serum bilirubin and was more prevalent in children whose total serum bilirubin was greater than to 34.2 μmol/L [1]. Serum bilirubin levels were also found to be inversely related to vitamin levels in a study by Shneider et al. [9]. Fat-soluble vitamins should be supplemented in all CPPs, and blood levels should be regularly monitored to adjust the dosage. No single multiple vitamin preparation is appropriate for all cholestatic infants; each child will need integration with individualized and unpredictable needs [10]. 

### 1.3. Routes of Administration

The treatment of fat-soluble vitamin deficiency is often complex in children with cholestasis, and how vitamin supplementation should be administered is still debated. Parenteral administration was suggested if bilirubin level exceeds 85 μmol/L, but high-dose oral formulations have been shown to be equally effective in some cases, unless deficiency refractory to oral therapy has been demonstrated [11]. The efficacy of a fat-soluble oral formulation (OAFSV) in pediatric patients with cholestasis was tested [4]. Among 23 pediatric patients recruited, the basal rates of vitamin A, D, E and K deficiency were 78.6%, 100%, 100% and 21.4%, respectively, in patients receiving standard treatment. After 3 months of oral supplementation, incidence of A, D and E deficiency had decreased; however, it was still present at 70.0%, 60.0% and 60.0% [4]. Thus, the standard oral supplementation is not safe and effective in preventing deficiencies without adequate monitoring of the individual response. Shneider et al. [9] evaluated the efficacy of a standard regimen of fat-soluble vitamin supplementation in a cohort of infants with BA after hepatoportoenterostomy (HPE). The prevalence of FSV deficiency in patients with failure of HPE (defined as persistent cholestasis after six months from surgery) was 100%, 79%, 50% and 46%, respectively, for vitamins A, D, E and K [9], thus demonstrating the ineffectiveness of these standard multivitamin preparations and the persistence of vitamin deficiency despite surgery. Therefore, careful monitoring is justified in children with cholestasis, particularly if caused by biliary atresia. Current strategies that have been shown successful include adding high-dose supplementation of a single vitamin, switching to more water-soluble forms or administering intramuscular injections. Multiple vitamin formulations are commercially available, but due to the variable deficiency rates of each vitamin in every patient, they are not indicated for cholestasis. Details of the dosage, intervals, and formulation of FSV administration used in patients with cholestasis change widely between studies and clinical recommendations. The fear of overdose or toxicity may lead to undertreatment of patients until the clinical and deficiency symptoms develop.

Table 2 summarizes the definitions of deficiencies and toxicities for each fat-soluble vitamin as reported in pediatric age [8,11,12,13] and by the ASPEN guidelines [14].

## 2. Materials and Methods

**Figure 1:**
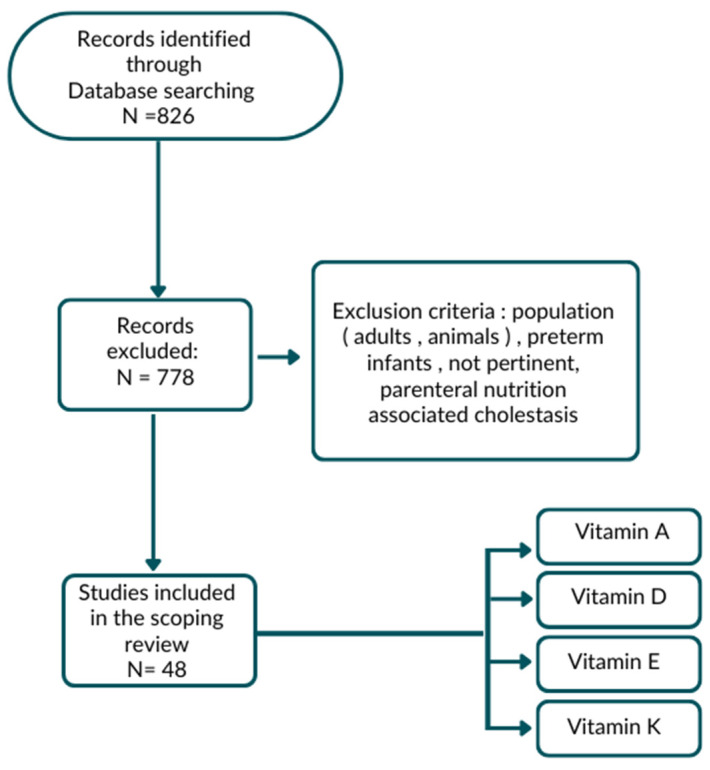
Flow chart of research strategy.

In consideration of the lack of data, the authors performed a comprehensive review of the literature for the last 20 years (up to February 2022) through the PubMed, Scopus, Web of Science and Embase databases. Keywords researched for each vitamin, alone or in combination, were: “cholestasis”, “chronic liver disease”, “biliary atresia”, “malnutrition” and “nutritional needs”. Two authors independently identified the most relevant studies, including original papers, narrative reviews, observational studies, clinical trials, systematic reviews and meta-analyses. Studies published prior to the selected time range were searched manually and, when considered relevant, included within the list of references. Studies in humans aged 0–18 years were included; studies in preterm infants or about parenteral nutrition-associated cholestasis were excluded (Figure 1). An analysis and comparison of the data in terms of incidence, methods of administration and recommended doses for the supplementation of fat-soluble vitamins in pediatric age was carried out.

## 3. Results

### 3.1. Vitamin A

The group of compounds known as retinoids, including retinol and its equivalents, are referred to as vitamin A. The synthesis of rhodopsin, a pigment required for retinal rod cells to operate and adapt to darkness and for proper cell differentiation, requires retinol [16]. From food sources, vitamin A is available in dairy, eggs and fish oils; as carotenoids (provitamin A), they are available from plants (leafy green vegetables, orange-colored fruits, vegetables). Vitamin A deficiency, documented in approximately one third of children with chronic cholestasis, can lead to symptoms such as dry skin, night blindness, xerophthalmia and keratomalacia [11,12,16]. Assessing the status of vitamin A can be accomplished using a variety of approaches. The most commonly used method is the assay of serum levels of retinol and/or retinol-binding protein (RBP); however, in pediatric hepatopatic patients, a definite correlation between circulating levels and liver stores of vitamin A has not been demonstrated [17]. Approximately, thirty-five children [17], including 23 with CCLDs, were studied by Feranchak to determine the validity of noninvasive methods for detecting vitamin A deficiency. The study suggested serum retinol level as an initial screen for this deficiency, followed by confirmation with a modified oral relative dose response (RDR) test. The RDR test reflects hepatic vitamin A stores after administration of a loading dose of vitamin A. When stores are normal, the plasma concentration of retinol does not change significantly. Plasma retinol concentration, however, significantly rises and peaks many hours after injection when hepatic vitamin A reserves are insufficient. This paradoxical effect is due to release of RBP from the liver in an attempt to redistribute absorbed vitamin A to peripheral tissues.

The indices of vitamin A deficiency were identified by serum markers:

- Serum retinol concentration <20 μg/dL (<0.693 μmol/L) (sensitivity 90%, specificity 78.2%);
- Serum retinol-binding protein (RBP) <1 mg/dL (sensitivity 40%, specificity 91.3%);
- Retinol/RBP molar ratio <0.8 mol/mol; sufficient level between 0.8 and 2.0 (sensitivity 60%, specificity 73.9%);
- IM relative dose response test (RDR): increase in plasma level of retinol by 20% 9 h after administration of an IM dose of vitamin A;
- Modified oral relative dose response (RDR) test: increase >20% 10 h after administration of 1.500 IU (450 μg) vitamin A as a water-soluble retinyl palmitate preparation administered orally with 25 IU/kg di TPGS (sensitivity 80%, specificity 100%).

Ocular complications can be assessed with

- Slit-lamp eye examination looking for signs associated with vitamin A deficiency, including xerosis, Bitot’s spots, keratomalacia and corneal ulceration;
- Tear film break-up time (TFBUT) represents the amount of time it takes for the first dry spot to form over the cornea and bulbar conjunctiva after applying a drop of 2% flour to the eye. Dry patches are denoted by black spots or lines. TFBUT denotes the time elapsed between the last blink and the onset of the first dry patch. TFBUTs shorter than 30 s are deemed abnormal;
- Schirmer test to measure tear production with a normal value >10 mm of wet paper;
- Conjunctival impression cytology (CIC).

Ophthalmologic evaluation is of little use as a screening method since the damage is late and unspecific; serum retinol was the most sensitive test (90%) and the modified oral RDR test the most specific (100%) [17]. 

Vitamin A supplementation can be completed orally or intramuscularly (IM) but requires careful monitoring, as hypervitaminosis A may be associated with neurological toxicity and hepatic damage [18]. Given the potential toxicity of vitamin A, beta–carotene supplementation can be considered as it is a precursor to vitamin A, and it is involved in protecting membranes against free radical damage. In vitro studies have also shown that the antioxidant effect of beta–carotene can alleviate the toxicity of bile acids on liver cells [19].

### 3.2. Vitamin D

The primary forms of vitamin D are vitamin D2 (ergocalciferol) and vitamin D3 (cholecalciferol), which are a group of fat-soluble prohormones and their metabolites [16]. Both forms are biologically inert and require two subsequent hydroxylations: first, in the liver, on carbon 25 to form 25-hydroxyvitamin D [25 (OH) D] and second, in the kidney, to form the biologically active form 1,25-dihydroxyvitamin D [1,25 (OH) (2) D]. Vitamin D is synthesized in the skin after exposure to sunlight or can be consumed in the diet from fish oils and fortified dairy products. [16,20]. In addition to being recognized as capable of lowering the risk of some diseases, vitamin D is crucial for the absorption of calcium and phosphorus, which are fundamental to maintaining healthy bones [20]. 

The causes of hypovitaminosis D include malabsorption, reduced dietary intake, reduced exposure to sunlight and impaired hepatic or renal hydroxylation. A deficiency of this vitamin is associated with hypocalcemia and hypophosphatemia, leading to reduced bone mineralization that, if left untreated, can led to osteomalacia and osteodystrophy with potential rickets and fractures [11,12,13,16,21,22]. 

The serum concentration of 25(OH)D is the strongest indication of vitamin D status in cholestasis [20]. The predominant form of vitamin D in serum, 25-OH vitamin D, has a half-life of two to three weeks and is made up of both dietary vitamin D intake and sun exposure generation. Conversely, 1,25(OH)2 D levels could be normal or even increased due to secondary hyperparathyroidism linked to vitamin D deficiency [20]. Although it is still debated [12], most experts concur that a 25-OH-D level of less than 20 ng/mL is “deficient” in vitamin D and that a level between 21 and 29 ng/mL is “insufficient” [11,20]. To fully benefit from all of the health advantages provided by vitamin D, it is important to keep both children and adults at a level >30 ng/mL [20].

Children with CLD have a very high prevalence of vitamin D insufficiency, with varying percentages as seen in Table 3. Children with bilirubin levels ≥34 μmol/L had significantly higher rates of vitamin D deficiency or insufficiency than children with bilirubin levels <34 μmol/L (47% vs. 19%; *p* = 0.028) [23]. Moreover, compared to the general population, patients with biliary atresia had a higher frequency of rickets and bone fractures [24]. Table 3 lists studies on the prevalence of hypovitaminosis D in children with cholestatic liver disease.

Reduced bone mineralization (BMD) is a pathological process that begins in early childhood, worsens rapidly with increasing age and liver dysfunction and remains relatively stable in children with chronic liver disease [28]. However, BMD and growth retardation in children with chronic cholestatic liver disease improve significantly after liver transplant [29,30,31,32].

Breastfed infants with CLD are especially vulnerable to the risk of BMD because their bone mineral content can decrease quickly during the first two years of life [33] and because breast milk contains low levels of vitamin D [16]. Cholecalciferol (D3), which is more soluble in water than other forms of vitamin D, should be preferred for administration. Compared to ergocalciferol (D2), this permits a higher bioavailability and affinity for the vitamin D-binding protein [34]. Children with cholestatic diseases were evaluated to determine the efficacy of high-dose oral vitamin D therapy (HDR), often known as “stoss therapy”, which had previously been tested in children with rickets and cystic fibrosis-related pancreatic insufficiency [35,36]. At the 4-week follow-up, all patients still had vitamin D deficiency (level 20 ng/mL). The findings imply that oral HDR therapy may not be a sufficient treatment for vitamin D shortage in children with cholestasis, in contrast to those without liver illness and those with pancreatic insufficiency [37].

A randomized controlled trial [38] compared the safety and efficacy of weekly regimens versus stoss therapy in children with CLD. Sixty-seven children with hypovitaminosis D were randomized to receive a “stoss regimen” (600,000 IU per day of oral cholecalciferol) or a weekly regimen (60,000 IU per week of oral cholecalciferol granules) of vitamin D3. Normal 25(OH)D levels at 6 months were achieved in 85.7% of those receiving the weekly regimen versus the 32.1% of those receiving the stoss regimen (*p* < 0.001), demonstrating the superiority of the weekly treatment compared to the “stoss regimen”. In addition, in eight patients with CPP, co-integration with a micellar vitamin E formulation (25 IU/kg) and vitamin D3 (1000 IU/kg) was found to boost vitamin D absorption in all patients, suggesting the combination of the two formulas as a possible best treatment strategy [39].

Table 4 lists the effectiveness of several treatment options for vitamin D insufficiency in children with cholestasis.

### 3.3. Vitamin E

Vitamin E, including tocotrienols and tocopherols, has important antioxidant properties and is found in nuts and seeds, vegetables and plant-based oils [16]. Vitamin E is transported by lipoproteins, and, consequently, cholestasis may be associated with an artificial increase in serum vitamin E levels [34,40]. Several authors agree that the ratio of serum vitamin E to total serum lipids (triglycerides, phospholipids, and total cholesterol) is the most reliable biochemical test of vitamin E status in patients with chronic childhood cholestasis [11,16,34,40]. The cut-off for vitamin E deficiency (VED) is 0.6 mg vitamin E/g total lipids in subjects aged from 1 to 12 years and 0.8 mg/g in older children and adults [12].

Chronic vitamin E deficiency may be associated with the onset of a progressive neurologic syndrome which can lead to irreversible damage [12]. It may cause nerve conduction disorders, such as peripheral neuropathy, ophthalmoplegia, ataxia, degenerative retinal lesions, and spino-cerebellar dysfunction, during the first decade of life. If uncorrected, it may impair walking by adolescence. In addition, vitamin E deficiency can also cause mild hemolytic anemia due to oxidative stress to red blood cell membranes and can adversely affect the immune system [41].

Several studies have shown that supplementation with fat-soluble forms of vitamin E (α-tocopherol, α-tocopheryl acetate, and α-tocopheryl succinate) is not indicated in cholestasis because they are not absorbed [42]. In a prospective, comparative longitudinal study [43], 60 vitamin E-deficient children with chronic cholestasis were divided into three groups assigned to different therapies of 100 IU, 200 IU and 400 IU/day of oral dl-α-tocopheryl acetate, respectively, for 15 days. The results reported that these dosages were not sufficient to maintain normal serum α-tocopherol concentrations [43]. Attempts to treat or prevent vitamin E deficiency with higher doses of oral α-tocopherol preparations (>100 IU/kg/die) were limited by the lack of intestinal bile acids impairing the formation of micelles for solubilization of α-tocopherol [44]. In patients with Alagille syndrome, standard treatment with α-tocopherol has also been shown to be insufficient [45] because plasma concentrations of lipid peroxides are much higher in these patients than in other forms of cholestasis. Intramuscular injections (IM) of α-tocopherol have been shown to reverse or prevent progression of neurologic symptoms if started promptly [46]. However, injections are painful and require frequent administrations. Sokol et al. studied and tested a new water-soluble oral form of vitamin E, D-α-tocopheryl polyethylene glycol 1000 succinate, tocofersolan (TPGS-E) [41]. This molecule consists of the binding, via a succinate ester bridge, of a polyethylene glycol (PEG) 1000 molecule to the end of D-α-tocopherol, thus creating an amphipathic structure that takes on a micellar form which allows it to cross the intestinal lumen and be absorbed by the enterocyte even in the presence of bile acid deficiency [42,47]. Clinical trials of TPGS-E were conducted in 22 children with severe cholestasis and vitamin E deficiency [47], demonstrating safety and efficacy in preventing and correcting vitamin E deficiency with no evidence of toxicity. Subsequently, the study was expanded to a multicenter study on 60 children [41], confirming the safety and efficacy of treatment with TPGS. The neurological impairment that had occurred in patients prior to the study stabilized or improved in 96% of patients. Therefore, it is possible to conclude that therapy with oral TPGS at a dose of 20–25 IU kg/day successfully prevents and corrects vitamin E deficiency in pediatric patients with chronic cholestasis, reversing, if not too advanced, the clinical signs of axonal neuropathy and ataxia, thus improving or stabilizing neurological function. These claims were also confirmed by a subsequent study [42] in 15 children with chronic cholestasis that investigates a possible effect of TPGS administration on lipid peroxidation levels induced by vitamin E deficiency, with the risk of inducing neuronal damage and reducing the availability of long-chain polyunsaturated fatty acids, which are essential for early neural development [42]. Treatment with TPGS corrected the serum levels of vitamin E; however, it did not improve PUFA deficiency or reduce lipid peroxidation, probably because of the progressive worsening of liver disease [42]. Roongpraiwan et al. [48] compared vitamin E state (plasma vitamin E/total lipids (E/L) ratio) in 11 children with biliary atresia during supplementation with 20 IU/kg/day, 100 IU/kg/day oral vitamin E (dl-α-tocopherol) or 50 IU/kg/day soluble form of vitamin E (CWSIF). Supplementation with either 20 IU/kg/day, 100 IU/kg/day vitamin E capsule, or 50 IU/kg/day vitamin E CWS/F corrected vitamin E levels in children who had serum direct bilirubin levels below 68.4 μmol/L but not in cases with serum direct bilirubin above 68.4 μmol/L [48]. The bioavailability of two oral formulations of vitamin E was evaluated in adult and children with chronic cholestasis or cystic fibrosis: the first treatment was with an oral formulation of 100 IU kg of reference vitamin E (vitamin E Cambridge containing dl-α-tocopheryl acetate) followed by an intermediate washout period of 1 week with a subsequent treatment with 2000 IU of tocofersolan (vitamin E TPGS Orphan Europe) [49]. Focusing on the group of six cholestatic children [49], the plasma concentrations remained very close to baseline values after administration of the reference vitamin E formulation. In contrast, vitamin E was well absorbed with a tocofersolan solution, confirming a significantly higher absorption and bioavailability of tocofersolan [49]. These data suggest tocofersolan as the preferable molecule in cholestatic children [41,47,49].

In 2009, the European Medicine Agency (EMA) [50] approved an oral liquid preparation of TPGS for the treatment of vitamin E deficiency in children with chronic cholestasis.

The drug was well tolerated, with no significant adverse reactions observed in 274 children with varied etiologies of chronic cholestasis, according to an analysis of the safety and effectiveness of TPGS treatment. There were only three minor reactions recorded, including one incidence of vomiting and two occurrences of abdominal pain that disappeared without intervention [51]. When considering efficacy analyses, treatment with 25 IU/kg of TPGS resulted in the majority of the vitamin E-deficient children (89%) regaining adequate vitamin E status at six months.

In addition, all children with previous normal baseline vitamin E levels maintained normal status at 6 months, as well as 93% of patients with unknown baseline levels [51]. Clinical studies on vitamin E supplementation are shown below in Table 5.

### 3.4. Vitamin K

The fat-soluble vitamin known as vitamin K is crucial for the maintenance of hemostasis, bone metabolism and the creation of sphingolipids in the brain. In the liver, vitamin K is required as a cofactor for the carboxylation of glutamic residues on coagulation factors II (prothrombin), VII, IX and X as well as proteins C and S [52]. In the absence of vitamin K, there is an increase in abnormal undercarboxylated forms of these proteins which are functionally ineffective, known as PIVKA-II (proteins induced in the absence of vitamin K) [53]. Therefore, the first consequence of vitamin K deficiency (VKD) is coagulopathy as a result of the altered balance of hemostasis. The inadequate carboxylation of osteocalcin, a bone matrix protein, determines bone disease [54] and increases the risk of fractures [53]. Three forms of vitamin K are known: Vitamin K1 (phylloquinone) is the main circulating form of vitamin K provided by food sources such as green leafy vegetables (spinach, brussels sprouts, cabbage, lettuce and broccoli). Vitamin K2 (menaquinone) is synthesized by intestinal flora and found in food products such as egg yolk, chicken, beef, liver and fermented cheese and vegetables. Vitamin K3 (menadione) is a synthetic form [55]. The level of vitamin K is evaluated using the prothrombin time (PT). The international normalized ratio (INR) is used to normalize the result. However, prothrombin (factor II) needs to be reduced to 50% before a change in PT occurs, proving that PT is an insensitive marker of vitamin K status [53]. Because they reflect current food consumption of vitamin K rather than reserves, vitamin K1 levels are also not sensitive indicators of vitamin K status [53]. A study of protein produced in the absence of vitamin K (PIVKA II) plasma levels is a more precise indicator: a value more than 3 ng/mL is suggestive of VKD. However, only a small number of laboratories offer the test, and it is rarely used in clinical settings [53].

Healthy newborns can easily develop VKD due to poor placental supply, low vitamin K content in breast milk and poor intestinal absorption due to immature intestinal flora, causing, if untreated, hemorrhagic disease.

Bleeding due to VKD in newborns is classified according to the time of onset:

- “Early” (within 24 h of life): It is rare and is typically seen in infants whose mothers have been prescribed drugs that interfere with vitamin K metabolism, either by known (oral anticoagulants such as warfarin) or uncertain mechanisms (anticonvulsants or antituberculous drugs, e.g., rifampin, isoniazid);
- “Classic” (within 1 week of birth): It is often considered idiopathic, but a known cause is inadequate nutrition and/or inadequate prophylaxis. Medications taken during pregnancy may also contribute;
- “Late” (>1 week and <6 months of age): It occurs when there is insufficient prophylaxis, in most cases this occurs in exclusively breastfed infants in whom there is co-morbid hepatobiliary cholestatic dysfunction that affects vitamin K malabsorption [52,55,56].

Whereas in healthy breastfed infants early neonatal hemorrhagic disease is prevented using vitamin K prophylaxis at birth, cases of late VKD-dependent bleeding continue to occur in infants with cholestasis [57] despite the prophylaxis given at birth. This serious complication may lead to a risk of 50–70% of intracranial hemorrhage (ICH), 20% mortality and 40% risk of severe neurologic sequelae. It can also present hematomas, gastrointestinal bleeding and bleeding from mucous membranes [58]. VKD is one of the earliest complications that develop in children with CLD, and the deficiency is directly related with its severity and cholestasis degree [53]. Since 1961, the American Academy of Pediatrics (AAP) recommends the administration of vitamin K prophylaxis at birth for the prevention of early, classic and late VKD bleeding (VKDB) [59,60]. In 2003, an IM injection of 1 mg of vitamin K at birth was proposed as the standard of care for healthy infants [61].

From national registries and incidence studies, it has become clear that the risk of late hemorrhagic disease is closely related to the presence of cholestasis, as illustrated below. In a survey conducted on the incidence and etiology of late VKDB in Dutch infants [62], five patients (83%) out of six reported cases of intracranial hemorrhage had cholestasis.

Medical records of 88 infants with biliary tract atresia (which received prophylactic oral vitamin K of 2 mg at birth) showed that 7 infants had ICH (7.95%); out of these, 4 patients were exclusively breastfed (BF), 1 patient was fed with formula milk supplemented with vitamin K, and 1 patient was fed both with breast milk and formula milk [63]. These data agree with findings from an observational study of children with ICH [64] which showed that more than 10% of patients had cholestatic liver disease.

The incidence of VKDB was prospectively followed over a 6-year period (2005–2011) by the Swiss Pediatric Surveillance Center [65], leading to an increase in the recommended dose from two to three oral doses (2 mg) during the neonatal period since [65] two doses were insufficient for avoiding late-onset VKDB. With the higher dose, only 1 early-onset VKDB case and 4 late-onset VKDB cases out of 458,184 live births occurred, for an overall incidence of 1.09/10 [56]. The late incidence of VKDB was 0.87/10 [56]. Moreover, all four children with late VKDB had undetected cholestasis, 3/4 of the parents had rejected VK prophylaxis, and 1/4 had neglected to take the third dosage of VK [66]. Thus, the main risk factors for VKDB in breastfed infants were parental VK prophylaxis refusal or unknown cholestasis [66].

Several therapeutic strategies have been proposed in cholestatic patients to prevent the risk of ICH. A prospective randomized controlled trial [67] studied the pharmacokinetics and efficacy of oral compared with intravenous (IV) vitamin K prophylaxis in infants with cholestatic liver diseases [67]: the first group received 1 mg IV while the second r group received 2 mg orally. Ninety percent of infants of the first group had elevated serum K1 at six hours, contrary to the second group, thus reflecting the better bioavailability of IV administration; however, this difference was significant only in the first 24 h.

Even though the oral administration of K1 may temporarily improve cholestasis-affected children’s vitamin K status, the amount absorbed is insufficient to build up long-term reserves. The effectiveness of a single intramuscular injection of vitamin K, on the other hand, has been hypothesized to determine a depot effect and a subsequent delayed release of the highly lipophilic vitamin from muscle tissue. An active surveillance for VKDB has been performed in Germany [68], and different formulations have been compared to the effectiveness of administration of oral vitamin K (2 mg) for three doses (2 mg at birth, 2 mg on days 3–10 and 2 mg in weeks 4–6). An incidence of late VKDB of 0.44/100,000 (95% CI 0.19–0.87) was reported in children given micellar vitamin K compared with 0.76/100,000 (95% CI 0.36 to 1.39) in children given other preparations (Cremoforo, polysorbate 80) [68].

In 43 children with CCLD, 54% of the children who received oral vitamin K supplements at the recommended dosages had vitamin K deficiency. This finding suggests that current dosing regimens, especially oral administration, are ineffective to prevent complications in this population [53].

Data from breastfed children who received the following prophylactic regimens were retrospectively analyzed from the Dutch and Danish national biliary atresia registries: 1 mg oral vitamin K at birth followed by 25 μg daily oral vitamin K prophylaxis (The Netherlands, 1991–2003); 2 mg oral vitamin K at birth followed by 1 mg weekly oral prophylaxis (Denmark, 1994–May 2000); and 2 mg intramuscular prophylaxis at birth (Denmark, June 2000–2005) or formula feeding (Supplementary Table S1) [69].

At the moment of biliary atresia’s diagnosis, the absolute and relative risk of severe VKD and cerebral bleeding were examined. Blood loss due to vitamin K deficiency was noted in 25 of 30 breastfed infants (83%) receiving 25 μg of daily oral prophylaxis, 1 out of 13 receiving 1 mg of weekly oral prophylaxis (7%), 1 out of 10 (10%) receiving 2 mg of intramuscular prophylaxis at birth and 1 out of 98 infants receiving formula (1%) (*p* < 0.001).

The relative risk of bleeding was higher (RR = 77.5) in the 25 μg daily oral prophylaxis’ group, suggesting that this regimen is not able to prevent bleeding in infants who had been misdiagnosed with cholestasis but appeared healthy [69].

These results indicate that breastfed infants with biliary atresia are not preserved by the Dutch prophylactic vitamin K regimen. At the time of cholestasis diagnosis, more than 80% of these kids had already had VKDB, and 43% of breastfed infants with biliary atresia experienced cerebral bleeding. Among Dutch breastfed infants with biliary atresia, the risk of VKDB was 8–10 times higher than in infants who had weekly oral prophylaxis or IM prophylaxis at birth; it was roughly 80 times higher than in infants who were formula-fed.

The oral 1 mg weekly regimen appears to be more efficient than the 25 μg daily regimen and is comparable to IM treatment (Supplementary Table S1). This is probably because of the dosage, as the cumulative dose in the weekly regimen was about five times higher (1.00 mg vs. 0.18 mg for weekly and daily oral prophylaxis, respectively). In children with cholestasis, this route of administration may be a useful substitute for IM treatment [69]. Formula feeding has been shown to be more protective for VKDB than IM prophylaxis in infants with unrecognized cholestasis. The mechanism remains unclear because the vitamin K content of formula (based on a daily formula intake of 150 mL/kg) is between 25 and 50 μg, similar to the dose prescribed in the Dutch prophylactic regimen.

Witt et al. [70] compared the efficacy of changing the Danish regimen with supplementation from 25 μg to 150 μg and the Danish regimen of a single intramuscular dose of 2 mg vitamin K at birth [70]. Data from children with BA were retrieved from national registries and the incidence of VKDB in the groups of children who received the three different supplementations was compared. VKDB occurred in 45 out of 55 (82%) infants in the 25 μg group, in 9 out of 11 (82%) in the 150 μg group but only in 1 out of 25 (4%) in the IM 2 mg group (*p* < 0.001) (Supplementary Table S2).

Cerebral bleeding was a presenting symptom in 27% of infants in the 150 μg group in comparison with the 40% of all infants in the 25 μg group (*p* = 0.43). The 2 mg IM group did not experience intracranial bleeding (0%; *p* < 0.001). In breastfed infants with undiagnosed BA, a preventive regimen of 1 mg of oral vitamin K at birth followed by daily oral doses of 25 or 150 μg failed to prevent VKDB. The data therefore confirmed the effectiveness of the prophylaxis against VKDB at birth with a 2 mg IM of vitamin K [70]. In conclusion, the ideal oral regimen of vitamin K supplementation to prevent late VKDB in newborns has not yet been determined using evidence-based data; however, it is clear that more than one single dose is required in the case of oral supplementation, particularly in exclusively breastfed infants. Unlike IM injections, oral administration of vitamin K may improve compliance and may even be more acceptable to some parents. Unpredictable absorption, unclear compliance and insufficient parental controls remain major disadvantages [55].

Although the pharmacokinetics of intravenous (IV) vitamin K are unknown, they are probably comparable to those of vitamin K taken orally. To prevent the late type of VKDB, intravenous delivery does not appear to be as effective as the IM route, particularly if the injection is not repeated [71].

According to the 2016 ESPGHAN Guidelines [55], healthy infants should receive one of the following prophylaxis modalities:

- 1 mg vitamin K1 by intramuscular (IM) injection at birth;
- 3 × 2 mg of vitamin K1 orally at birth, at 4–6 days and at 4–6 weeks;
- 2 mg of vitamin K 1 orally at birth and a weekly dose of 1 mg orally for 3 months.

In terms of effectiveness and dependability, IM administration is the most popular approach globally. Oral supplementation has been proposed, but it is not indicated in premature infants, infants with cholestasis or intestinal disorders and infants whose mothers have taken medications that may interfere with vitamin K metabolism. Thus, the best VKDB prophylaxis for this group of infants is currently thought to be a rapid postnatal IM vitamin K treatment [55]. We reported in Table 6 the studies found with the comparison of dose, route of administration and efficacy demonstrated for supplementation of vitamin K.

## 4. Discussion

Our review shows that the treatment of fat-soluble vitamin deficiency in children with cholestasis is complex, and each patient requires a tailored approach. Meanwhile, in clinical practice, it is often necessary to start from an empirical treatment, considering that serum levels of fat-soluble vitamins are not always immediately available, and laboratory results often require prolonged technical times. For this reason, in Table 7 we compare the indications for treatment, according to different authors and guidelines.

Most of these reviews have been focused on the nutritional indications for cholestasis and do not analyze exclusively the treatment of vitamin deficiencies. Considering data from the literature and the comparison of clinical studies and reviews, we propose an adapted summary of the treatment strategies for each vitamin (Table 8).

## 5. Conclusions

Our scoping review shows that pediatric patients with cholestasis have a high risk of fat-soluble vitamin deficiency. However, although there are general guidelines on management of these patients, there are still evidence gaps about dosages and supplementation strategies. The indications provided in the literature are not currently validated or clearly homogeneous, neither in terms of dosages nor in terms of routes of administration. Our study compared the different indications that can be used initially in the supplementation of patients, but it remains necessary to individually adjust the treatment according to plasma vitamin values. The complex management of these patients requires new studies to validate the efficacy of commercially available formulations and to improve new effective formulas.

## Figures and Tables

**Table 1:** Incidence of fat-soluble vitamin deficiency in pediatric cholestatic patients.

Population	FVS Deficiency	Reference
Children with BA compared with non-cholestatic children	Vit. A: 77.2% Vit. E: 50.9% Vit. D: 37.6%	Saron et al., 2009 [2]
166 children with end-stage liver disease, including 126 CPP (75%)	Vit. A: 66.6%, Vit. E: 40.6% Vit. D: 36.3%	Veraldi et al., 2019 [3]
23 pediatric cholestatic patients	Vit. A: 73.9%, Vit. E: 91.3% Vit. D: 81.8% Vit. K: 20.0%	Shen et al., 2012 [4]
266 pediatric patients with obstructive jaundice	Vit. A: 15.2%, Vit. E: 3.9% Vit. D: 87.8% Vit. K: 5.4%	Dong et al., 2017 [5]

BA = biliary atresia; FSV = fat-soluble vitamins.

**Table 2:** Evaluation, deficiency and toxicity of fat-soluble vitamins (A, D, E and K) in pediatric cholestatic liver disease; adapted from [14,15].

Vitamin A	Deficiency	Toxicity	Normal Levels
Measurement	Retinol/RBP ratio < 0.8 Serum RBP < 1 mg/dL Serum retinol levels < 20 µg/dL Modified oral relative dose response (RDR) test with increase > 20% after administration of vit A	Difficult to assess biochemically	Retinol 0–6 months: >20 µg/dL 6 months: 30–80 µg/dL
Clinical signs	Dry skin, xerophthalmia, night blindness, Bitot’s spots, keratomalacia, anorexia, anemia, leucopenia, hyperkeratosis, depressed helper T cell activity and/or impaired mucus secretion	Hepatic and neurologic toxicity, long bone fractures, muscle and bone pain, cheilitis, pseudotumor cerebri, photophobia, alopecia, ataxia, conjunctivitis, hepatotoxicity and hyperlipidemia	
Vitamin D			
Measurement	Serum 25-OH-D: <20 ng/mL: vitamin D deficiency; <30 ng/mL: vitamin D insufficiency.	Excess > 100 ng/mL Intoxication: >150 ng/mL)	Optimal level: 30–40 ng/mL
Clinical signs	Hypocalcemia/hypophosphatemia/tetany, osteomalacia and rickets	Hypercalcemia leading to depression of the central nervous system and ectopic calcification. Hypercalciuria leading to nephrocalcinosis Weakness, fatigue, diarrhea, anorexia, headache, confusion, psychosis, tremor	
Vitamin E			
Measurement	Vit E/total lipid ratio Deficiency of vit E: <0.6 mg/g (age < 1 year) <0.8 mg/g (age > 1 year)		Serum α-tocopherol >0.7 mg/dL
Clinical signs	Hypo- or a-reflexia ataxia, hemolytic anemia, impaired vibratory sensation, proximal muscle weakness, ophthalmoplegia, degenerative lesions of the retina	Worsening of vit K deficiency coagulopathy Diarrhea Hyperosmolality (TPGS)	
Vitamin K			
Measurement	Prothrombin time International normalized ratio Protein induced in vit K absence II (PIVKA II): <3 ng/mL		Prothrombin time (11–15 s) PIVKA II > 3 ng/mL
Clinical signs	Hemorrhage	No major toxicity	

**Table 3:** Studies on the prevalence of hypovitaminosis D in children with cholestatic liver disease.

Subjects	Deficiency (D) and Insufficiency (I)	Rickets	Fractures	Low Bone Mineral Density	Author
N = 50 Controls (30)	Hypovitaminosis 56% D = 30% (N = 15) I = 26% (N = 13)	N = 28 (56%)	N = 6 (12%)	N = 19 (67.8%)	Samra et al., 2018 [13]
N = 59 (N = 17 (30%) with bilirubin ≥34 μmol/L)	Hypovitaminosis 28% D = 14% (N = 8) I = 14% (N = 8)	N = 13 (22%)			Lee et al., 2018 [23]
N = 48	Hypovitaminosis 64% D = 10.4% (N = 5) I = 54.1% (N = 26)	N = 22 (45.8%)			Mohammad et al., 2012 [25]
N = 22 Controls 17	Hypovitaminosis(<9 ng/mL) = 36%				Bastos e da Silveira, 2003 [26]
N = 49	Hypovitaminosis 64% D = 10.4% (N = 5) I = 54.1% (N = 26)	N = 7 (14%)	N = 11 (72%)		Ruuska et al., 2020 [24]
N = 92	Hypovitaminosis 98.9% D = 81.5% (N= 75) I = 17.4% (N = 16)				Ng et al., 2016 [27]

D = deficiency, I = insufficiency.

**Table 4:** Studies on treatment of hypovitaminosis D in children with cholestatic liver disease.

Vitamin D Supplementation	Population	Comments	
High-dose oral vitamin D therapy (HDR) “stoss therapy”: 300,000 IU ergocalciferol	32 children with cholestasis	All patients remained in deficient of vitamin D.	[37]
Comparison of weekly regimens versus stoss therapy	67 children with CLD and hypovitaminosis D	Increased 25(OH)D levels with weekly regimen.	[38]
Cointegration of vitamin D and micellar vitamin E formulation	8 children with severe chronic cholestasis	Increased absorption of vitamin D when administered with TPGS.	[39]

**Table 5:** Studies on the management of hypovitaminosis E in children with cholestatic liver disease.

Study	Population	Comments	Author
Evaluation of 3 different dosages of oral dl-alpha-tocopheryl-acetate: 100 IU/day; 200 IU/day; 400 IU/day.	60 vitamin E-deficient children with chronic cholestasis	Therapy safe but not effective in maintaining levels of vitamin E.	Velasco Benitez et al., 1996 [43]
Efficacy of IM vitamin E injections (10 mg/kg dl-α-Tocopherol acetate) every 2 weeks (max 200 mg) Evaluation of oxidative stress in cholestatic patients with Alagille syndrome	15 children with AGS Group 1: plasma bilirubin <100 μmol/L (*n* = 6); Group 2: plasma bilirubin >100 μmol/L (*n* = 9); Control: 15 healthy children.	IM administration of α-T (at a dose of 10 mg/kg every 2 weeks) is insufficient in patients with Alagille syndrome to prevent lipid peroxidation.	Davit-Spraul et al., 2001 [45]
Safety and efficacy of the TPGS form on vitamin E deficiency	22 children (aged 7 months to 19 years) with severe cholestasis and vitamin E deficiency	Safe and effective form of vitamin E for the prevention and correction of vitamin E deficiency during childhood cholestasis.	Sokol et al., 1987 [47]
Therapy with 20–25 IU kg/day of oral TPGS	60 children with chronic cholestasis	Oral TPGS therapy prevents and corrects vitamin E deficiency, replacing IM injections.	Sokol et al., 1993 [41]
TPGS therapy on lipid peroxidation levels	15 children with chronic cholestasis deficient in vitamin E	Rapid normalization of serum vitamin E concentrations but no improvement on PUFA deficiency on lipid peroxidation.	Socha et al., 1997 [42]
Supplementation of 20 IU/kg/day oral capsule of vit E ((dl-alpha-tocopherol); 100 IU/kg/day; 50 IU/kg/day vitamin E (CWSIF).	11 children with biliary atresia	All 3 therapies were effective in normalizing vitamin E status in cholestatic children with direct bilirubin levels <68.4 μmol/L. With direct bilirubin >68.4 μmol/L, none of the vitamin E supplementation corrected the deficiency.	Roongpraiwan et al., 2002 [48]
Bioavailability of two oral formulations of vitamin E REFERENCE: 100 IU/kg Vit. E (dl-α-tocopheryl acetate miscible in water); TEST: 100 IU/kg water-soluble tocopherolol (TPGS).	6 children with biliary atresia and total bilirubin >100 μmol/L	The absorption and bioavailability of oral tocofersolan was significantly greater than the reference formulation in children with chronic cholestasis.	Jacquemin et al., 2009 [49]
Safety/efficacy of D-α-tocopheryl polyethylene glycol 1000 succinate (Tocofersolan, Vedrop). Daily dose of 0.34 mL/kg (25 IU/kg or 17 mg/kg) body weight.	274 children with cholestasis, including 142 biliary atresia; 38 Alagille syndrome; 39 neonatal transient cholestasis; 26 PFIC; 13 metabolic diseases; 6 cystic fibrosis; 10 other causes.	Safe and effective form of vitamin E that quickly restores and/or maintains adequate serum levels of vitamin E in most children with cholestasis, avoiding the need for intramuscular injections.	Thébaut et al., 2016 [51]

**Table 6:** Studies on prevention of VKDB in children with cholestatic liver disease.

Vitamin K Supplementation	Study Population	Comments	Author
Comparison of pharmacokinetics and efficacy of mixed oral and intravenous micellar prophylaxis	44 infants with cholestatic liver disease Group 1: 1 mg K by intravenous injection; Group 2: 2 mg oral.	Poor overall absorption in cholestatic infants with insufficient levels to provide long-term protective storage.	Pereira et al. [67], 2003
Efficacy use of oral mixed micellar vitamin K3 × 2 mg (2 mg at birth, 2 mg on days 3–10 and 2 mg in weeks 4–6) compared with other preparations		Slightly lower incidence of late VKDB by 0.44/100,000 (95% CI 0.19–0.87) in children on mixed micellar vitamin K.	Von Kries et al. [68], 2003
Prevalence of vitamin K deficiency with oral supplementation according to standard dosing guidelines	43 children with chronic cholestatic liver disease	Vitamin K deficiency prevalence of 54%; current regimens are not effective for cholestatic patients.	Mager et al. [53], 2006
Comparison of risk of vitamin K deficiency bleeding with different prophylactic regimens: 1 mg oral vit K at birth + 25 μg oral prophylaxis/day; 2 mg oral vit K at birth followed by 1 mg oral prophylaxis/weekly; 2 mg IM at birth	142 infants with biliary atresia, including 53 breastfed and 89 formula-fed	Risk of VKDB 8 to 10 times greater in breastfed infants with daily prophylaxis compared with weekly oral or IM prophylaxis at birth and nearly 80 times greater compared with formula-fed infants. Weekly 1 mg prophylaxis is more effective than 25 μg daily prophylaxis and is similar to IM administration.	Van Hasselt et al. [69], 2008
Incidence and long-term outcome of ICH in patients with BA who previously received prophylactic vitamin K	Medical records of 88 infants with BA	All patients received prophylactic oral vitamin K (2 mg) during the neonatal period, 7 had ICH (7.95%).	Alatas et al. [63], 2012
Efficacy of 2 oral doses of vitamin K (2 mg)	475,000 births	18 cases of late VKDB, including 13 with persistent liver disease. Two oral doses of 2 mg of a mixed micellar vitamin K preparation failed to prevent VKDB.	Schubiger et al. [65], 2003
VKDB incidence after modification of Swiss guidelines, 2 to 3 oral doses (2 mg)	458,184 born alive	4 cases of late VKDB; all had undiagnosed cholestasis at the time of bleeding. 3/4 had refused prophylaxis and 1/4 had forgotten the third dose.	Laubscher et al. [66], 2013
Compared efficacy of 3 regimens: 1 mg oral at birth + 25 μg/day; 1 mg oral at birth + 150 μg/day; single IM dose of 2 mg at birth.	91 breastfed babies with BA	A prophylactic regimen of 1 mg oral vitamin K at birth followed by oral/daily dose of 25 or 150 μg fails to prevent VKDB in neonates. Single IM administration is effective.	Witt et al. [70], 2016

**Table 7:** Comparison of recommended standard dosages for the administration of fat-soluble vitamin (A, D, E and K) deficiency in pediatric cholestatic liver disease.

A	D	E	K	Author
Oral: <10 kg 5000 IU/day; >10 kg 10,000 IU/day. IM: 50,000 IU monthly	Oral: 25-OHD: 80–200 IU//kg/die IM: 30,000 IU 1–3 monthly	Oral: TPGS 25 IU/kg IM: 10 mg/kg (max. 200 mg) every two weeks	Oral: 2 mg/day weekly 5 mg: 5–10 kg; 10 mg: >10 kg; IM: 5–10 mg every two weeks.	Baker et al., Guidelines 2007 [11]
5000–20,000 IU	80–160 IU/kg/day	15–25 mg/day	2400–15,000 μg/day	ASPEN 2010 [14]
			1 mg vitamin K1 IM at birth; 3 × 2 mg oral vitamin K1 at birth, at 4–6 days and at 4–6 weeks; 2 mg of vitamin K 1 orally at birth and a weekly dose of 1 mg orally for 3 months.	Guidelines ESPGHAN 2016 [55]
5000–10,000 IU/day	Sunlight exposure; vitamin D-1-α 50 ng/kg	TPGS: 50–400 IU/day	2.5–5.0 mg/day	Kelly et al. [7] 2003, Review
<10 kg: 5000 IU; >10 kg: 10,000 IU. IM: 50,000 IU	25-OH-D: 80–200 IU/kg/die IM: 30,000 IU, 1–3 monthly	TPGS 25 IU/kg IM: 10 mg/kg (max. 200 mg) every 3 weeks	Oral: 2 mg/day Week: 5–10 kg: 5 mg; >10 kg: 10 mg; IM: 5–10 mg every 2 weeks.	Socha, 2008 [12], Review
5000–25,000 IU/g	400 IU/day, in 25-OH D3 form (in association with TPGS to improve absorption)	TPGS: 15–25 IU/kg/day	2.5–5 mg/day	Nightingale et al., 2009 [21], Review
1000 IU/kg/day to 25,000 IU (Oral) <10 kg: 5000 IU/day; >10 kg: 10,000 IU/day.	Cholecalciferol: Weight > 40 kg and serum level: <10 ng/mL: 5000 IU/day; 11–19 ng/mL: 4000 IU/day; 20–29 ng/mL: 3000 IU/day. Weight < 40 kg 120–200 IU/kg/day	TPGS: 15–25 IU/kg/day	2.5–5.0 mg 2 to 7 times a week. 1–10 mg (intravenous if necessary)	Yang et al., 2017 [16], Review
<10 kg 5000 IU/day; >10 kg 10,000 IU/day.	Cholecalciferol: 2000–5000 IU/day	TPGS: 15–25 IU/kg/day	2–5 mg/ day	Mouzaki et al., 2019 [34], Review
3000–10,000 IU/day <10 kg: 5000 IU/day; >10 kg: 10,000 IU/day. IM: 50,000 IU/1–3 monthly	Oral: Cholecalciferol: 800–5000 IU/day 1.25-OH Cholecalciferol: 0.05–0.2 µg/kg/day	Oral: Alpha-tocopherol acetate: 15–25 to 25–200 IU/kg/day TPGS: 15–25 IU/kg	2.5–5.0 mg/day from twice a week to every day Oral: 5–10 kg: 5 mg; >10 kg: 10 mg. IM: 5–10 mg/day every two weeks.	Tessitore et al., 2021 [15], Review

IM, intramuscular injection; TPGS, D-α-tocopheryl polyethylene glycol 1000 succinate; IU, international units.

**Table 8:** Proposal of initial dosage for treatment of fat-soluble deficiency.

	Dosage	Route of Administration
Vitamin A	<10 kg 5000 IU/day; >10 kg 10,000 IU/day [12]	Oral
Vitamin D	Cholecalciferol 80–200 IU/kg/die oral, 30,000 IU 1–3 monthly IM [11,12]	Oral, IM
Vitamin E	TPGS 25 IU/kg/die [41,47]	Oral
Vitamin K	<5 kg: 1 mg/kg every 2 weeks; >5 kg: 10 mg every 2 weeks [11,12]	IM

## Data Availability

Not applicable.

## Supplementary Materials

The following supporting information can be downloaded at: https://www.mdpi.com/article/10.3390/nu15112491/s1, Table S1: Comparison of risk of vitamin K deficiency bleeding (VKDB) in infants with biliary atresia with different prophylactic regimens; Table S2: Comparison of vitamin K deficiency bleeding (VKDB) in different prophylactic regimens in breastfed infants with biliary atresia.
