# Supplementary material for: Fat-Soluble Vitamins Deficiency in Pediatric Cholestasis: A Scoping Review
Source: Nutrients. 2023 May 26;15(11):2491. doi: 10.3390/nu15112491 (PMC10255381; doi:10.3390/nu15112491)
Supplement: Supplementary file 1 [file nutrients-15-02491-s001.zip › nutrients-2351429-supplementary.pdf]

Supplementary Table S1. Comparison of risk of vitamin K deficiency bleeding (VKDB) in infants with biliary atresia with different prophylactic regimens.

	**Netherlands, 1991-2003**	**Denmark, 1994- May 2000**	**Denmark, June 2000-2005**
Breastfed	1 mg oral vit K at birth + 25 μg daily oral prophylaxis<br>N= 30<br>(VKDB) n= 25	2 mg oral vitamin K at birth followed by 1 mg weekly oral prophylaxis<br>N= 13<br>(VKDB) n= 1	2 mg intramuscular prophylaxis at birth<br>N= 10<br>(VKDB) n= 1
Fed with formula	N= 98<br>(VKDB) n= 1		
Relative risk of bleeding in breastfed versus formula-fed infants	RR= 77,5	RR= 7,2	RR= 9,3

Supplementary Table S2. Comparison of vitamin K deficiency bleeding (VKDB) in different prophylactic regimens in breastfed infants with biliary atresia

**Netherlands, 01/1991-02/2011**	**Netherlands, 03/2011-01/2015**	**Denmark, 07/ 2000-11/2014**
1 mg oral vit K at birth + 25 μg daily oral prophylaxis<br>N= 55<br>(VKDB) n= 45 (82%)	1 mg of oral vitamin K at birth followed by 150 μg of daily oral prophylaxis<br>N= 11<br>(VKDB) n= 9 (82%)	2 mg intramuscular prophylaxis at birth<br>N= 25<br>(VKDB) n= 1 (4%)

VKDB= bleeding depending from vitamin K deficiency
